# Sustainable production of housefly (*Musca domestica*) larvae as a protein-rich feed ingredient by utilizing cattle manure

**DOI:** 10.1371/journal.pone.0171708

**Published:** 2017-02-07

**Authors:** Mahmoud Hussein, Viju V. Pillai, Joshua M. Goddard, Hui G. Park, Kumar S. Kothapalli, Deborah A. Ross, Quirine M. Ketterings, J. Thomas Brenna, Mark B. Milstein, Helene Marquis, Patricia A. Johnson, Jan P. Nyrop, Vimal Selvaraj

**Affiliations:** 1 Department of Animal Science, College of Agriculture and Life Sciences, Cornell University, Ithaca, New York, United States of America; 2 Division of Nutritional Sciences, Cornell University, Ithaca, New York, United States of America; 3 Johnson Graduate School of Management, Cornell University, Ithaca, New York, United States of America; 4 Department of Microbiology and Immunology, College of Veterinary Medicine, Cornell University, Ithaca, New York, United States of America; 5 Department of Entomology, New York State Agricultural Experiment Station, Cornell University, Geneva, New York, United States of America; Institute of Zoology Chinese Academy of Sciences, CHINA

## Abstract

The common housefly, *Musca domestica*, is a considerable component of nutrient recycling in the environment. Use of housefly larvae to biodegrade manure presents an opportunity to reduce waste disposal while the rapidly assimilated insect biomass can also be used as a protein rich animal feed. In this study, we examine the biodegradation of dairy cattle manure using housefly larvae, and the nutritional value of the resulting larva meal as a feed ingredient. Our results demonstrated that dairy cattle manure presents a balanced substrate for larval growth, and the spent manure showed reductions in concentration of total nitrogen (24.9%) and phosphorus (6.2%) with an overall reduction in mass. Larva yield at an optimum density was approximately 2% of manure weight. Nutritional analysis of *M*. *domestica* larva meal showed values comparable to most high protein feed ingredients. Larva meal was 60% protein with a well-balanced amino acid profile, and 20% fat with 57% monounsaturated fatty acids, and 39% saturated fatty acids. Larva meal lacked any significant amount of omega-3 fatty acids. Evaluation of micronutrients in larva meal suggested that it is a good source of calcium and phosphorus (0.5% and 1.1% respectively). The nutritional value of larva meal closely matches that of fishmeal, making it a potentially attractive alternative for use as a protein-rich feed ingredient for livestock and aquaculture operations.

## Introduction

Livestock generate important sources of protein for human consumption. Conversely, manure generated by livestock represents a primary management challenge in areas with high-density livestock production. More than 335 million tons of manure (in dry matter) is produced annually on farms in the US [1]. In areas with a high animal density and limited options for export, total manure generation can be in excess of that can be safely applied to agricultural land as fertilizer [2], contributing to eutrophication of water bodies, contamination of ground water, and threats of disease [3]. In most cases, the costs associated with manure treatment and export have implications that percolate all the way to the consumer [4].

In parallel, increasing global demand for livestock and aquaculture products to satisfy the growing human population has introduced new facets to environmental and food security concerns. Feed ingredients, which represent approximately 60–70% of livestock production costs, is emerging as a major challenge to economically increase production. Some common staple food resources between humans and animal production systems instigate both competition, and pressure on cultivatable land; estimates indicate that livestock already account for 70% of all agricultural land use [5]. In addition, need for high nutritional value animal-based protein sources like fishmeal for aquaculture, poultry and swine operations has resulted in expansion of commercial fishing that is not sustainable [6]. In 2010, it was calculated that 73% of fishmeal and 71% of fish oil produced were consumed by aquaculture operations [7]. Exploitation of ocean resources has led to adverse ecological impact reducing wild fish stock and modifying oceanic habitats [8, 9]. Therefore, there is an ongoing quest for sustainable high quality ‘alternate’ protein ingredients that reduce both environmental impact and costs associated with livestock production.

Irrespective of environmental concerns, there is also pressure for identifying alternate protein rich feed ingredients that could replace feeding of mammalian meat and bone meal to cattle, sheep and goats to avoid risk of prion diseases such as bovine spongiform encephalopathy (BSE) [10]. However, the required inclusion of animal-sourced protein that is of high biological value in aquaculture diets compared to plant-based sources [11, 12], and in high-quality poultry diets [13, 14], has left very few options for complete alternates.

As early as the 1930s, anticipation of food demand in the period between the world wars triggered exploration into insects as food [15]. More systematic work in the 1970s and 80s, highlighted some of the distinct potentials of using specific species of insect larvae in aquaculture and livestock production [16–21]. However, there was little impetus for a commercial “larva meal” feed enterprise at that time because fishmeal was abundantly available and cost effective. With recent estimates that food production needs to almost double to host 9 billion people by 2050 [22–24], there is imminent need to re-evaluate livestock production systems for sustainability and improved efficiency with minimal waste generation and environmental impact. Edible insects are fast biomass generators with exceptional value as both feed and degraders of waste [25, 26]. As a result, several recent studies have revisited specific species of insects as a feed ingredient for different livestock operations [27–32].

Many species of Diptera complete their life cycle naturally in animal wastes. The concept that housefly (*Musca domestica*) can be used to convert waste to high protein feedstuff has been considered for decades [17]. Perhaps, the idea of rearing larvae on dairy cattle manure did not carry customer appeal to the general public when first considered, or the process and undesirable accidents of flies escaping could have been a deterrent [33]. At the present time, with increasing demand and the advances in technologies for automating biological production systems, it seems appropriate to reopen this topic of investigation and plan for its commercial potential.

In this study, we conducted experiments to assess the feasibility of using *M*. domestica reared on dairy cattle manure as an animal feedstock. We examined larval development, survival and behavior as a function of larval density in manure, manure degradation and the nutritional content of larvae reared on manure.

## Materials & methods

### Housefly colony

Adult houseflies captured in proximity to the Cornell Teaching Barn were used to establish breeding colonies. Fly cages (50 x 50 x 50 cm; BugDorm, MegaView Science, Taiwan) were used to house adult flies. Approximately 750 houseflies (emerging from pupae) were kept in each cage for a period of 3 weeks for each breeding cycle. After this period, cages were cleaned and disinfected for fresh young breeders. Water in cages was provided using containers that had protruding cotton wicks as drinking sites. A mixture of powdered milk and granulated sugar (1:1 ratio) was provided *ad libitum* in open containers as food for the flies. Cages were located in a room with a photoperiod of 12h light and 12h darkness, temperature of 25 ± 2°C, and ambient humidity (40–60%).

### Egg production

From approximately 5 days after emergence, flies were provided with oviposition substrate in the form of 100 g of cattle manure in a 100 mm petri dish. Flies were allowed to oviposit for a 4-hour period every 48 hours over 3 weeks. Eggs were separated using a modified suspension method [34]. In brief, egg masses were transferred to a 0.8M sucrose solution, agitated and buoyant eggs were collected from the top layer. Eggs were then washed in water three times to remove any impurities and sucrose. The eggs were finally rinsed using a 75 μm stainless steel filter (EmCon Filter, Agtech, Inc., USA), briefly air dried, and integrity was evaluated under the microscope prior to weighing. Eggs were suspended in water (5 mg eggs/ml, approximately 75 eggs) to facilitate distribution immediately before use.

### Manure and larvae handling

Fresh dairy cattle manure was collected from the Cornell Teaching Dairy Barn directly from the floor and transported to the laboratory for immediate experimentation, or stored at 4°C and used over 2–3 days maintaining its 85% moisture content. All larval growth/migration and manure degradation experiments were performed in an environmental chamber (H: 250 cm x W: 300 cm x L: 300 cm) under a regulated temperature of 28 ± 2°C, relative humidity of 60–70%, and photoperiod of 12h ambient light and 12 h darkness. Egg hatching, larvae growth and behavior were monitored without removing the larvae from this chamber. Incubation and analyses were dependent on experimental objectives. For larva meal nutritional analysis, the egg suspension, as prepared above, was evenly sprinkled over the manure; 50 mg (10 ml of suspension) was used for 250 g of manure in plastic containers (H: 8 cm x W: 12 cm x L: 30 cm). Larvae were harvested before pupation at the third instar approximately 5–6 days of growth and were rinsed using water, dried, and stored at -20°C until analysis.

### Larvae density and growth

The effect of egg seeding density on housefly larvae total harvest weight was estimated using defined quantities of manure and eggs. Freshly collected manure samples (25 g) were incorporated with different densities of eggs (1, 2, 4, 8 or 16 eggs/g of manure). Hatching and growth conditions were monitored and final harvest weight (both individual and total) after 5 days of larval growth were measured. Average larva weight was calculated by dividing the total harvest weight by the number of larvae harvested. Survival rate was calculated as the number of harvested larvae relative to the number of eggs incorporated in manure samples. By allowing the larvae to pupate (8–9 days), pupation rate was evaluated for the different egg incorporation densities.

### Larvae migration

Larva migration within the manure mass during development was assessed using clear plastic containers (H: 15 cm x W: 8 cm x L: 8 cm). Within this container, manure covering a depth of 10 cm was overlaid with approximately 150 eggs and allowed to hatch. Migration of larvae within the column of manure was monitored over the period of larval development. The deepest points reached by larvae from the surface were measured by following the burrowing traces visible along the wall of the container. This depth was averaged across three experimental containers.

To examine the rate of larvae outmigration/egress with increasing density of egg incorporation, samples (25 g) of freshly collected manure were incorporated with different densities of larvae (1, 3, 5 and 7, 1-day-old larvae/g manure). Larva growth and outmigration was observed during 7 days of subsequent growth and pupation. Outmigration rate was calculated as the number of larva exiting the manure over the lip of the container at 5, 6 and 7 days relative to the number of larvae incorporated in the manure.

### Manure degradation

To test the effect of larvae density on manure degradation, manure (25 g) was incorporated with 0 (control), 1, 2, 4, 8 or 16 eggs/g manure. Five days later, larvae were harvested/removed and manure was collected, dried, and weighed. Manure degradation was calculated as the percentage change in manure mass in each group relative to change in the control group.

Evaluation of changes to manure composition as a result of growing larvae were performed using larvae numbers considered optimal based on results from studies on density and growth. Manure (25 g) in containers were provided 1-day-old larvae (3 larvae/g manure), covered with a piece of gauze and kept in the environmental chamber. After 7 days, larvae (and some pupae) were harvested and the manure collected, weighed and analyzed for composition. Manure treated the same way without larvae were collected as controls.

### Proximate analysis

Composition of manure and larvae were analyzed using standard methods at Brookside laboratories (New Bremen, OH, USA). Samples were oven-dried for determination of dry matter content (according to AOAC #930.15). Total nitrogen (N) content was determined using combustion analysis on the Elementar Vario max CN instrument (Elementar Analysensysteme, Hanau, Germany; according to AOAC #990.03). Total protein was estimated from N content using a standard factor of 6.25 (according to AOAC #990.03). Ash content was determined using a muffle furnace with samples subjected to 600°C and analysis (according to AOAC #942.05). Crude fiber (CF), acid detergent fiber (ADF) and neutral detergent fiber (NDF) were determined according to AOAC methods #962.09, 973.18 and 2002.04, respectively. Crude fat was determined through ether extraction and analysis (according to AOAC Modified #920.39).

### Mineral analysis

Mineral content of dried manure samples was estimated by first subjecting them to nitric acid and hydrogen peroxide digestion using a microwave-based solvent extraction system (MARS, CEM Corporation, Mathews, NC, USA). Calcium, phosphorus, potassium, magnesium, sodium, sulfur, iron, manganese, copper and zinc contents were analyzed using inductively coupled plasma mass spectrometry (Thermo 6500 Duo ICP, Waltham, MA, USA).

### Amino acid analysis

Samples were analyzed for the different essential amino acids (EAA: tryptophan, methionine, cysteine, lysine, threonine, arginine, isoleucine, leucine, valine, histidine, and phenylalanine) and nonessential amino acids (NEAA: glycine, serine, proline, alanine, aspartate, and glutamate). The analyses of amino acids were performed using previously described methods [35]. In brief, three hydrolysis procedures using hydrochloric acid, or performic acid oxidation prior to hydrochloric acid, or using barium hydroxide, were performed in duplicate for each sample. Amino acids were then separated using a lithium cation exchange column (4 x 100 mm, P/N 0354100, Pickering Laboratories, Mountain View, CA, USA) with a three-buffer step gradient (Li292, Li365 and Li375, Pickering Laboratories, Mountain View, CA) and a column temperature gradient (33, 42, 60 and 70°C); detection was at 560 nm following ninhydrin reaction post column derivation on an HPLC System Gold with 32 Karat software (Beckman-Coulter, Inc., Fullerton, CA, USA).

### Fatty acid analysis

Samples (200 mg) were homogenized and lipids were extracted using a standard protocol [36]. Extracts were methylated overnight at 40°C in 1% methanolic sulfuric acid and subsequently transmethylated [37], using a modified protocol as described [38]. The resulting fatty acid methyl esters (FAME) were dissolved in heptane and stored at -20°C until analyses. FAME were quantitatively analyzed using a HP 5890 series II GC-FID with a BPX 70 column (length: 60 m, inner diameter: 0.32 mm, film: 0.25 μm; Hewlett Packard, Palo Alto, CA, USA) with H_2_ was used as a carrier gas, and a GC-flame ionization detector and structural identification by gas chromatography-covalent adduct chemical ionization tandem mass spectrometry (GC-CACI-MS/MS) as previously described [39–42]. An equal weight FAME mixture (68A; Nu-Chek Prep, Inc., Elysian, MN, USA) was used to calculate response factors on a daily basis [41]. All GC analyses were performed in triplicate.

### Fish oil supplemented manure

In order to test if altering the fatty acids available in manure could change the endogenous fatty acid composition of larvae that were particularly deficient in omega-3 fatty acids, menhaden fish oil preparation containing the omega-3 polyunsaturated fatty acids (EPA and DHA at 30%; Arista Industries Inc, Wilton, CT) was mixed with manure (1 ml/25 g fresh manure) and used to grow larvae at a density of 1 larva/g manure for 5 days in the environmental chamber as described. Larvae grown in manure (control), and fish oil enriched manure (treatment), were then harvested, cleaned and homogenized for lipid extraction and fatty acid analysis as described (n = 6/group). Relative changes to the different fatty acids were evaluated to examine for the changes in omega-3 fatty acid content in housefly larvae.

## Results and discussion

Larval stages of the housefly life cycle are completed in different forms of organic materials. Fly control on dairy farms has been linked to manure management [43], suggesting that manure waste might be a good medium for growing larval stages of housefly larvae. Few studies have examined this potential from the standpoint of fly biology. Freshly dropped dairy cattle manure has inherent physical conditions (pH 5–6 and osmolality 400–500 mOsm/Kg) that are known to favor immediate oviposition and colonization of this fly species [44]. The houseflies that emerge from pupae after completing their larval stages in cattle manure appear to have optimal fertility, fecundity and longevity [45], suggesting that this substrate is able to support breeding. Despite their benefits in decomposing organic materials, flies have become a problem of public health in farms [46–48]. Exploiting this naturally tuned lifecycle of houseflies in manure could be of both environmental and economic benefit to animal agriculture. In this study, we focused on identifying optimal parameters and criteria that would allow for the scaling-up of larva meal production for use by the feed industry.

### Manure composition

Intensive housefly larvae rearing necessitates that cattle manure should meet levels of nutrition to meet the need for different larval stages at densities higher than that seen in the wild. Similar to higher animals, it has been documented that insects have essential nutrient requirements for optimum growth and development [49, 50]. Previous studies have showed that swine manure [27] and poultry manure [17, 51] could support housefly larvae growth at fairly high densities. However, manure composition differs between species based on diet composition and gut physiology. Therefore, it was important to examine the nutrient profile of dairy cattle manure available for the development of housefly larval stages, in comparison to previous reports for swine and poultry manure.

Proximate analysis of fresh dairy cattle manure (Table 1), showed a dry-matter:moisture ratio of approximately 1:5.6. The dry manure had a crude protein content of 15.3% and crude fat content of 1.2%. Total metabolizable energy (ME) for manure was estimated as 2.22 Mcal/kg of manure. Crude fiber content was 33.5%, as expected for a roughage-based ruminant diet. This composition was different from values reported for swine and poultry manure; crude protein content of cattle manure was lower than averages reported for swine manure (19%) and chicken manure (23.3%) [52, 53]. Previous studies indicate that the above crude protein level measured for dairy cattle manure is subject to variations in type of ration; it was previously reported that crude protein levels in dry dairy cows averaged 11.4%, in lactating dairy cows averaged 19.7%, in heifers averaged 12.2% and in beef cattle averaged 17.6% [52]. Although overall crude protein values for dairy cattle manure are typically lower than that of chicken, and pig manure, they were certainly not prohibitive for larval growth and development. The ability of cattle manure to support housefly larvae growth and development also indicated that the necessary levels of carbohydrates, essential fatty acids, vitamins and minerals were available in cattle manure. Moreover, there was no indication of toxic effects due to undesirable constituents or nutritional imbalance in cattle manure. Interestingly, total digestible nutrients (TDN) for dairy cattle manure was estimated as 61.6% (Table 1), a value that was very comparable to chicken broiler manure of 68.9% [53], suggesting that cattle manure may indeed contain valuable nutrients for housefly larvae growth and pupation.

**Table 1:** Nutrient composition of cattle manure.

Composition^1^	Manure
Dry Matter (%)	15.09 ± 0.70
Crude Protein (%)	15.29 ± 1.00
Fat (%)	1.20 ± 0.09
Fiber (%)	33.45 ± 1.70
Ash (%)	24.25 ± 3.16
TDN (%)	61.62 ± 1.39
ME (Mcal/Kg)^2^	2.22 ± 0.02
Calcium (%)	2.07 ± 0.29
Phosphorous (%)	0.68 ± 0.08
Potassium (%)	0.75 ± 0.08
Magnesium (%)	0.64 ± 0.07
Sodium (%)	0.39 ± 0.02
Iron (ppm)	1054.83 ± 137
Manganese (ppm)	137.70 ± 3.55
Copper (ppm)	46.17 ± 2.05
Zinc (ppm)	170.83 ± 12

^1^ All values are reported on 100% DM basis or ppm.

^2^ ME value were calculated from DE values (ME = 0.82 X DE)

### Intensive rearing of larvae on manure

**Fig 1:**
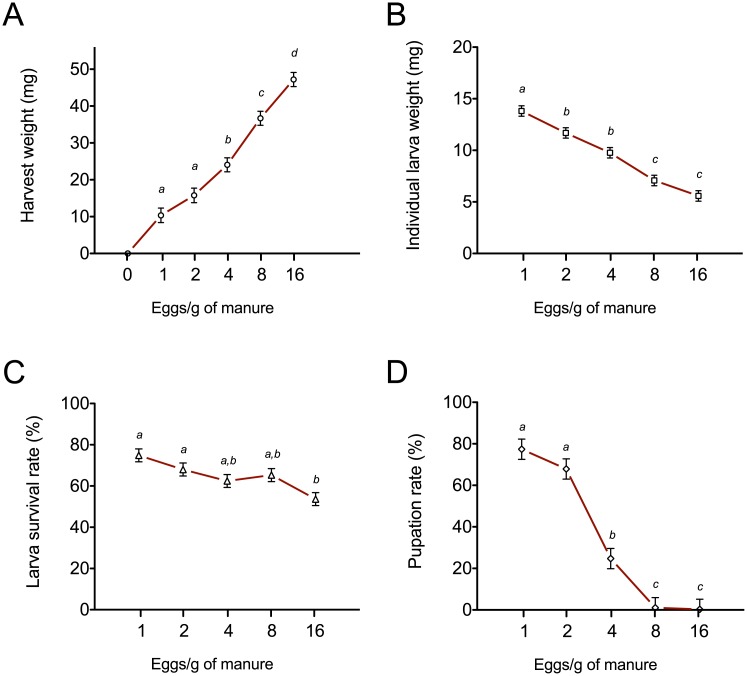
Effects of intensive rearing on housefly larvae growth and development. Twenty-five gram manure samples were incorporated with 1, 2, 4, 8 or 16, housefly eggs/gram manure (n = 7). Eggs were allowed to hatch and larvae were harvested after 5 days of growth. For the different larvae densities: (A) Harvest weight was determined by weighing all harvested larvae at the end of the experiment; (B) Individual larva weight was calculated by dividing the total harvest weight by the number of harvested larvae’ (C) Larva survival rate was calculated by dividing the number of harvested larvae and/or pupae by to the number of eggs incorporated; (D). Pupation rate was calculated by dividing the number of harvested larvae and/or pupae by the number of eggs incorporated. Values represent means ± SEM. Groups not connected by same letter are significantly different (*P* < 0.05).

Manure from cattle proved to be an excellent oviposition substrate attracting adult houseflies to lay eggs when introduced into the fly cages. This fly behavior in and of itself was a good indication that cattle manure could support larval development. The housefly eggs hatched, and the larvae completed their three instars in manure, pupated and emerged as adult flies. The manure-cultured flies reproduced for generations without any problems. In manure incubated in the environmental chamber at 28°C, the period from hatching to pupation took approximately 8 days. This duration can be dramatically affected by temperature changes that have a negative correlation with the total life cycle of this species [54]. Manure supported increasing densities of eggs for subsequent larvae development of up to 16 eggs/gram of manure. After 5 days of growth, total harvest weights showed a density-dependent increase (Fig 1A) suggesting that higher density of egg incorporation could lead to higher larvae yield. However, individual larva weights showed a negative correlation with each increase in egg incorporation density (Fig 1B) from 13.8 mg in 1 egg/g of manure to 5.5 mg in 16 egg/g of manure. Evaluation of larva survival rate indicated that densities up to 8 eggs/g manure caused no significant problems with survival; at 16 eggs/g of manure the survival rate was significantly decreased (53.6%) compared to higher survival (74.8 and 68% respectively) at low densities of 1 or 2 eggs/g of manure (Fig 1C). Pupation rate indicated that the higher density egg incorporations of 8 or 16 eggs/g manure did not result in successful pupation (Fig 1D) suggesting that the health and viability of larvae/pupae were compromised at these higher densities. Even the few pupae that were obtained at these high densities did not result in emergence of adult flies. This result could be due to three overlapping possibilities: (1) nutrients to support larval growth in manure could become limiting at high densities, (2) generation of metabolic byproducts could lead to an unfavorable change to the manure microenvironment, and/or (3) space available for foraging for each larva could be limiting at high densities. From the above experiments on intensive rearing of larvae, we conclude that cattle manure contains sufficient nutrients to support the life cycle of houseflies at low densities. Based on the different densities used, 4 eggs/g of cattle manure is considered appropriate for larvae growth and acceptable harvest weights for larva meal production (this density was chosen for nutritional analysis of larva meal). For propagating breeder flies, lower densities of 1 or 2 eggs/g of cattle manure are essential to ensure optimal health and fertility.

### Larvae migratory behavior

**Fig 2:**
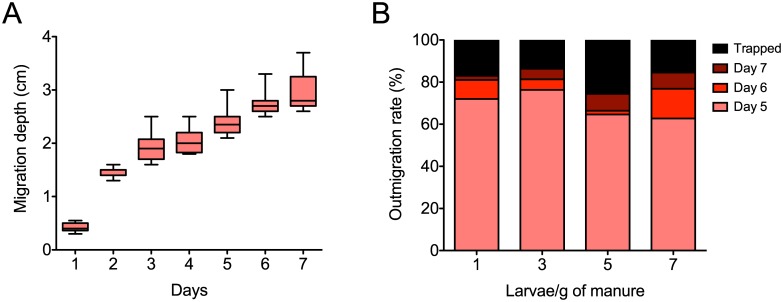
Relationship between density and migratory behavior of housefly larvae in dairy cattle manure. (A) Larva migration was observed over the 7 days of growth. Migration depth was calculated as the distance within manure (cm) that larvae were able to reach during the experiment. Data are represented as medians, 25^th^ to 75^th^ percentile as hinges, and range as whiskers. (B) Larvae outmigration rate was calculated as the percentage of larvae exiting or ultimately trapped in the manure environment at days 5, 6 and 7 of growth. This rate was compared across different densities of incorporation (1, 3, 5 and 7 larvae/g of fresh manure; n = 4/group).

Migration of larvae within the manure is an indicator of foraging behavior, and provides means to assess the extent of manure utilization in a three dimensional space. Migration depth of larvae measured over a 7-day growth period indicated that the larvae explored deeper as they got older reaching up to 3 cm from the surface (Fig 2A). It remains unclear if this is an activity change, or a need to forage in previously unused manure as nutrients become limiting at the surface over time. Foraging depth could also be proportional to increase in the physical size of larvae that increases with age. This experiment provided valuable input for designing large-scale production systems regarding the depth of manure needed for optimal usage/degradation by larvae. The density of larvae incorporation in manure did not have a significant influence on the outmigration rate, an indicator of larval egress mainly for pupation. At a density of 1 larva/g of manure, the outmigration rate was 83%, and at 7 larvae/g of manure it was 84.6% over a period of 7 days. This suggested that irrespective of competition for space or nutrient needs, the larvae remain in the manure until they are ready for pupation. This behavior also indicated that at higher larvae densities, biodegradation of manure could be greater.

### Manure biodegradation

**Fig 3:**
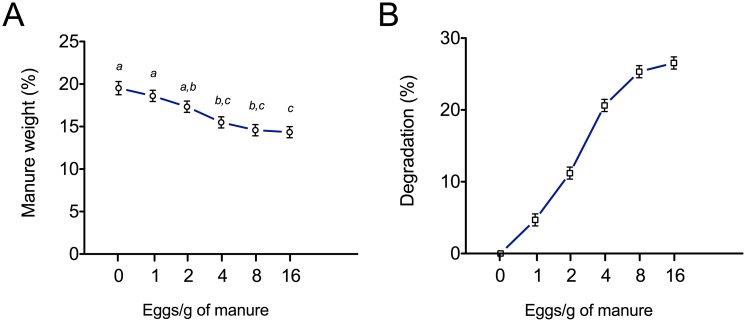
Dairy cattle manure degradation resulting from housefly larvae growth. (A) Housefly eggs incorporated in 25 g of fresh dairy cattle manure at different densities (0, 1, 2, 4, 8 or 16 eggs/g) were allowed to hatch and grow (n = 7/group). Decrease in manure weight after growth for 5 days was measured after harvesting larvae for the different groups. (B) Manure degradation rate was calculated as the percentage change in manure weight for each egg density group relative to the change in the control group after 5 days of larvae growth. Values represent mean ± SEM. Groups not connected by same letter are significantly different (*P* < 0.05).

In addition to supporting growth of larval stages, an important synergy anticipated with use of manure in larva meal production is degradation and mass reduction of the manure. Results from experiments examining percentage change in manure weight with increasing larvae incorporation density showed that manure degradation increased asymptotically to a maximum with increasing larval density (Fig 3A). At the density of 1, 2, 4, 8 and 16 eggs/g of manure, manure mass decreased by 0.9%, 2.2%, 4.1%, 4.9% and 5.2% respectively. These values were after taking into account the loss of moisture during the 5-day experimental period (control: 0 eggs/g of manure). Percentage decreases in manure mass compared to the control were 4.7%, 11.2%, 20.6%, 25.3%, and 26.5% at densities of 1, 2, 4, 8, and 16 eggs/g manure (Fig 3B). The relative plateauing of manure degradation at higher larvae densities could reflect depletion of ingredients of nutritional value to larvae. This suggested that larvae densities above 16 eggs/g manure would not further deplete manure mass. In confirmation of this assumption, use of spent manure after larvae harvest to re-incorporate eggs failed to support larval growth (data not shown).

Changes to manure composition specific to plant nutrients as a result of growing larvae for a period of 7 days are presented in Table 2. Results indicated that larva growth decreased total nitrogen content by 24.9%, the majority of which was a decrease in organic nitrogen (19.8%). Larva growth also decreased phosphorus content by 6.2%. There was a relative increase in calcium and magnesium levels after larva growth. Mineral matter and total organic matter were not significantly changed as a result of larvae growth. These results suggested that housefly larvae mainly utilized proteins present in the manure. Studies on foraging behavior of housefly larvae have indicated that they feed on microbes [55]; this suggests that larvae could be consuming microbes that grow in the manure, and use little of the manure itself. Therefore, larvae grown in manure may be dependent on the microbial ecosystem that exists, or is being established during the period of larval growth. There is already an extensive gut microbial ecosystem in freshly defecated manure that would offer an excellent substrate for larvae growth. However, vigorous consumption and depletion of the microbes by larvae would prevent different forms of microbial degradation from occurring in the manure (particularly for carbohydrates). Unsustainable microbial depletion could self-limit the potential for more complete manure usage. Therefore, kinetics of microbial populations and timing of housefly egg incorporation are areas that may need further investigation to maximize degradation potential and larvae yield.

**Table 2:** Effect of larva growth on manure composition.

Composition^1^	Control	With larvae	Change	%Difference
Mineral Matter (%)	34.59±1.65	32.81±1.38	-1.78	- 5.2%
Organic Matter (%)	65.41±1.65	67.19±1.38	1.78	+ 2.6%
Total Nitrogen (%)	2.69±0.06	2.02±0.05	-0.67	- 24.9%
- Ammonia-N (NH_4_-N) (%)	0.17±0.05	*Not detected*	*-*0.17	- 100%
- Nitrate-N (NO_3_-N) (%)	>0.01±0.00	>0.01±0.00	0	0%
- Organic-N	2.52±0.09	2.02±0.05	-0.5	- 19.8%
Phosphorus (%)	0.81±0.02	0.76±0.05	-0.05	- 6.2%
Potassium (%)	0.93±0.01	0.93±0.03	0	0%
Calcium (%)	2.27±0.02	4.42±1.99	2.15	+ 48.6%
Magnesium (%)	0.71±0.01	0.90±0.08	0.19	+ 21.1%
Sodium (%)	0.47±0.01	0.47±0.02	0	0%
Sulfur (%)	0.35±0.01	0.34±0.01	-0.01	- 2.9%
Boron (ppm)	16.10±0.17	16.57±1.07	0.47	+ 2.8%
Iron (ppm)	1493.33±50	1550.00±117	56.67	+ 3.7%
Manganese (ppm)	165.33±3.28	156.00±7.00	-9.33	- 5.6%
Copper (ppm)	87.37±50	81.50±3	-5.87	- 6.7%
Zinc (ppm)	209.67±30	187.33±17	-22.34	-10.7%

^1^ All values are reported on 100% DM basis.

### Proximate analysis of larva meal

Nutrient composition of housefly larvae in comparison to known values for soybean meal and fishmeal is presented in Table 3. The results confirmed that larva meal had all the qualities to be classified as high protein feed ingredient containing 59.9% crude protein. This proportion of crude protein in larva meal is comparable to reported values for fishmeal (65.3% crude protein) and higher than reported values for soybean meal (49.4% crude protein). Interestingly, we observed that larva meal is nutrient dense with a high fat content of 19.6%, a value 2-fold higher than fishmeal (10.2%), and 22-fold higher than soybean meal (0.9%). Total metabolizable energy (ME) for larva meal at 2.97 Mcal/kg was identical to values reported for fishmeal (3.07 Mcal/kg), and higher than values reported for soybean meal (2.51 Mcal/kg) [56]. Based on the analyzed values for crude protein, fat and ME, we conclude that larva meal can be improved substitute for soybean meal and very comparable to fishmeal as a feed ingredient.

**Table 3:** Nutrient composition of larva meal compared to soybean and fishmeal.

Composition^1^	Larva meal	Soybean^2^	Fishmeal^3^
Dry Matter (%)	24.30±0.43	88.2	92.1
Crude Protein (%)	59.87±1.31	49.4	65.3
Fat (%)	19.64±1.10	0.90	10.22
Fiber (%)	7.11±0.24	7.87	0.76
Ash (%)	7.06±0.32	*-na-*	*-na-*
NFE (%)	6.32±0.45	*-na-*	*-na-*
TDN (%)	82.40±1.21	*-na-*	*-na-*
ME (Mcal/Kg)^4^	2.97±0.044	2.51	3.07
Calcium (%)	0.49±0.01	0.33	5.55
Phosphorus (%)^5^	1.09±0.02	0.735	3.13
Potassium (%)	1.27±0.02	2.25	0.71
Magnesium (%)	0.23±0.02	0.31	0.17
Sodium (%)	0.54±0.02	0.03	0.71
Iron (ppm)	475±166	90	478
Manganese (ppm)	274±30	34	36
Copper (ppm)	32.4±6.5	18	12
Zinc (ppm)	1039±37	28	160

^1^All values are reported on 100% DM basis.

^2^ Soybean meal, solvent extracted, international feed #: 5-40-604. Nutrient Requirements of Poultry: Ninth Revised Edition, 1994

^3^ Menhaden fish, international feed #: 5-20-009. Nutrient Requirements of Poultry: Ninth Revised Edition, 1994

^4^ ME for larvae meal and pupae meal were calculated from DE values (ME = 0.82 x DE)

^5^ This is total phosphorus. Soybean meal has 0.30% non-phytate phosphorus

Abbreviations used are NFE, nitrogen-free extract; TDN, total digestible nutrients; ME, metabolizable energy.

### Mineral content of larva meal

Mineral composition of larva meal is presented in comparison to soybean meal and fishmeal in Table 3. Calcium and phosphorus content of larva meal were higher than that observed in soybean meal, but much lower than values observed in fishmeal. Magnesium levels in larva meal were comparable to those of soybean meal, and higher than fishmeal. Fishmeal contains a proportion of fishbone that greatly increases calcium and phosphorus values. Phosphorus levels in larva meal were 2-fold higher than calcium levels, an opposite trend compared to fishmeal. Phosphorus is a mineral that needs to be supplied in feed, compared to calcium that can be obtained directly from the water by several commercial fish species. Fishmeal contains 3-fold higher phosphorus levels compared to larva meal. Calcium and phosphorus utilization from both fishmeal and larva meal in diets need to be interpreted with caution as bioavailability can limit use and result in elimination. Much of the phosphorus in commercial fish diets, due to fishmeal use, may be released into the environment [57], negatively impacting water quality. Therefore, it is possible that use of larva meal in fish feed formulations could be highly beneficial to meet protein requirements without releasing excessive phosphorus into water bodies.

Levels of the different micro-minerals evaluated (iron, manganese, copper and zinc) in larva meal showed values higher and more balanced than that found in soybean meal, and except for iron, in fishmeal as well. These are also positive points for use of larva meal as a feed ingredient.

### Amino acid composition of larva meal

The percentage of protein and protein quality are the most important aspects to be considered for feed protein sources. One criterion to define protein quality in an ingredient is the balance of amino acids. The nitrogen content and amino acid composition of larva meal compared to soybean meal and fishmeal are presented in Table 4. The amino acid profile of larva meal was better balanced than soybean meal and fishmeal with no limiting amino acids. When compared for essential amino acids, larva meal had higher content of methionine, phenylalanine and tyrosine compared to both soybean meal and fishmeal. Methionine content of larva meal was 5.7- and 2.3-fold higher than values reported for soybean meal and fishmeal respectively. Phenylalanine content of larva meal was 1.6-fold higher than values reported for both soybean meal and fishmeal. Tyrosine content of larva meal was 1.6- and 1.8-fold higher than values reported for soybean meal and fishmeal, respectively. Larvameal also contained taurine similar to other animal sourced proteins including fishmeal [58]. Content of Isoleucine in larva meal was 0.8- and 0.7-fold, modestly lower than values reported for soybean meal and fishmeal respectively. Other amino acids were mostly similar between larva meal and soybean meal. However, compared to fishmeal, larva meal contained modestly higher levels of tryptophan (1.3-fold) and cysteine (1.3-fold), and modestly lower levels of leucine (0.7-fold) and lysine (0.8-fold). These results indicate that the amino acid profile in larva meal proteins is well balanced and that inclusion of larva meal in rations of livestock and fish would not require supplementation.

**Table 4:** Amino acid composition of larva meal compared to soybean and fishmeal.

Composition^1^	Larva meal	Soybean^2^	Fishmeal^3^
Dry matter	25.80±0.77	*-na-*	*-na-*
Nitrogen	0.11±0.003	*-na-*	*-na-*
***Amino acids***			
Alanine	2.91±0.05	*-na-*	*-na-*
Arginine	3.17±0.05	3.56	4.00
Aspartic acid	5.18±0.22	*-na-*	*-na-*
Cystine	0.78±0.01	0.75	0.62
Glutamic acid	8.38±0.07	*-na-*	*-na-*
Glycine	2.22±0.05	2.15	4.84
Histidine*	1.59±0.03	1.33	2.57
Isoleucine*	1.84±0.06	2.22	2.48
Leucine*	3.28±0.03	3.84	4.52
Lysine*	3.85±0.05	3.05	4.90
Methionine*	4.03±0.09	0.70	1.77
Phenylalanine*	3.82±0.13	2.45	2.40
Proline	2.49±0.06	*-na-*	*-na-*
Serine	2.25±0.03	2.60	2.57
Taurine	0.26±0.01	*-na-*	*-na-*
Threonine*	2.49±0.01	1.95	2.67
Tryptophan*	0.69±0.03	0.84	0.53
Tyrosine	3.51±0.15	2.17	1.95
Valine*	2.59±0.06	2.35	3.01

*na*–not available

^1^All values are reported on 100% DM basis.

^2^ Soybean meal, solvent extracted, international feed #: 5-04-604. Nutrient Requirements of Poultry: Ninth Revised Edition, 1994

^3^ Menhaden fish, international feed #: 5-20-009. Nutrient Requirements of Poultry: Ninth Revised Edition, 1994

* Essential amino acids

### Fatty acid composition of larva meal

The proportions of saturated and unsaturated fatty acids are important for determination of lipid quality of a diet. Dietary fatty acids can have significant influence on body fat composition [59, 60]. The different fatty acids identified in larva meal and their relative concentrations are presented in Table 5. The composition of fatty acids seen in larva meal was unique with 57.5% of monounsaturated fatty acids [contributed largely by C16:1n-7 (33.31%) and C18:1n-9 (21.22%)]. Saturated fatty acids ranked second with 38.64% [mainly contributed by C16:0 (25.28%)]. Polyunsaturated fatty acids made 3.86% [mainly contributed by C18:2n-6 (2.82%)]. Omega-3 fatty acids represented less than 1% of the overall profile, with alpha linolenic acid [C18:3n-3 (0.56%)] being the main contributor to this category.

**Table 5:** Fatty acid composition of larva meal.

Fatty acids	Larva meal^1^
13:0	1.56±0.18
14:0	3.85±0.21
Iso 14:0	0.65±0.03
14:1	2.03±0.09
15:0	2.82±0.15
Iso 15:0	0.19±0.01
Anteiso-15:0	1.83±0.13
16:0	25.28±0.07
Iso 16:0	0.04±0.01
16:1n-7	33.31±0.43
16:2	-nd-
16:3	-nd-
Iso 17:0	0.00±0.00
18:0	2.41±0.14
Iso 18:0	-nd-
18:1n-9	22.22±0.82
18:2n-6, LA	2.82±0.06
18:3n-6	-nd-
18:3n-3, ALA	0.56±0.01
18:4n-3	0.39±0.02
20:0	-nd-
20:1	-nd-
20:4n-6	0.05±0.01
20:5n-3, EPA	-nd-
22:1	-nd-
22:5n-3	-nd-
22:6n-3, DHA	-nd-
24:5n-3	-nd-
24:6n-3	-nd-
SAT	38.64±0.38
MUFA	57.50±0.93
PUFA	3.86±0.30
n-6	2.91±0.06
n-3	0.95±0.02

nd—not detected (values less than 0.05)

^**1**^ Values represent percentages of fatty acids (means ± SEM).

Little work has been done on aspects of fatty acid synthesis in housefly larvae. First, it was identified that sterols are essential for housefly larva survival [61]. It was subsequently elucidated that larvae can synthesize all necessary fatty acids, as they could subsist on a casein-based diet supplemented with sterols [62]. However, from the above results, it remained unclear if larvae directly assimilated some fatty acids from the manure. In order to test if changing fatty acids in the growth environment could modify housefly larval fatty acid profile, we tested if integrating some fish oil into manure could increase omega-3 fatty acid levels in larva meal. Our results showed that providing fish oil changed larvae fatty acid composition (Table 6); it increased the proportion of polyunsaturated fatty acids by 4.2-fold (from 3.86 to 16.07%). This increase in polyunsaturated fatty acids was mainly in the form of 20:5n-3 (EPA; 8.72%) and 22:6n-3 (DHA; 0.76%). Relative to the increase in polyunsaturated fatty acids, monounsaturated and saturated fatty acids dropped by 0.8-fold (from 57.5 to 48.53%) and 8.4% (from 38.64 to 35.41%) respectively. These results indicate that waste substrates used for larva meal production could influence the fatty acid composition of the resulting larva meal.

**Table 6:** Changes to larva meal fatty acid composition when grown in fish oil supplemented manure.

Fatty acids	Enriched larva meal^1^^,^^2^	Change	Fold change	Fish oil^1^^,^^3^
13:0	0.93±0.39	- 0.63	0.6	2.57±0.10
14:0	4.47±0.16	0.62	1.2	2.90±0.14
Iso 14:0	0.19±0.03	- 0.46	0.3	0.04±0.01
14:1	0.86±0.05	- 1.17	0.4	0.10±0.03
15:0	1.16±0.06	- 1.66	0.4	0.15±0.04
Iso 15:0	0.12±0.02	- 0.07	0.6	2.10±0.06
Anteiso-15:0	0.66±0.05	- 1.17	0.4	0.07±0.02
16:0	23.77±0.47	- 1.51	0.9	7.02±0.42
Iso 16:00	-nd-	-0.04		0.11±0.03
16:1n-7	23.61±0.35	- 9.70	0.7	5.24±0.02
16:2	0.79±0.02	0.79	∞	0.64±0.16
16:3	0.31±0.02	0.31	∞	0.58±0.03
Iso 17:00	-nd-		0.0	5.35±0.01
18:0	4.02±0.23	1.61	1.7	1.57±0.07
Iso 18:00	-nd-		0.0	0.38±0.01
18:1n-9	20.99±0.72	- 1.23	0.9	16.03±0.28
18:2n-6, LA	3.38±0.07	0.56	1.2	2.64±0.02
18:3n-6	0.09±0.03	0.09	∞	0.00±0.00
18:3n-3, ALA	0.86±0.05	0.30	1.5	0.70±0.03
18:4n-3	0.37±0.02	- 0.02	0.9	0.38±0.02
20:0	-nd-		0.0	0.15±0.04
20:1	2.84±0.02	2.84	∞	3.25±0.04
20:4n-6	0.65±0.02	0.60	13.0	2.37±0.03
20:5n-3, EPA	8.72±0.33	8.72	∞	29.55±0.27
22:1	0.23±0.03	0.23	∞	0.89±0.03
22:5n-3	0.12±0.02	0.12	∞	1.29±0.03
22:6n-3, DHA	0.76±0.03	0.76	∞	13.56±0.77
24:5n-3	-nd-			0.06±0.02
24:6n-3	-nd-			0.05±0.02
SAT	35.41±0.68	- 3.23	0.9	22.41±0.47
MUFA	48.53±0.80	- 8.97	0.8	25.77±0.28
PUFA	16.07±0.35	12.21	4.2	51.82±0.84
n-6	4.92±0.08	2.01	1.7	5.65±0.17
n-3	11.14±0.34	10.19	11.7	46.17±0.82

nd—not detected (values less than 0.05)

^**1**^ Values represent percentages of fatty acids (means ± SEM)

^**2**^ From manure supplemented with fish oil

^**3**^ Menhaden fish oil

### Conclusions

Identifying alternate sources of protein ingredients for use in livestock feed is an important means of securing resources for sustaining the growing human population and the associated global food demand [63]. There is accumulating evidence that use of insect larvae as a protein-rich feed ingredient can fill this need due to inherent benefits that include rapid production rate, ability to use waste products, high nutritional value, and reduction in overall agricultural land use for livestock [64]; unique benefits to growth and pathogen resistance have also been reported [65]. Moreover, rapidly growing global livestock operations like aquaculture that is projected to exceed beef, pork and poultry production within the next decade [66], is in dire need of sustainable feed resources for maintaining this growth pattern. By examining larvae growth characteristics, manure biodegradation potential, and its nutrient content as a protein-rich feed ingredient, this study paves the way towards development of a much-needed industrial larva meal-based livestock feed enterprise.

This study represents the first detailed examination of intensive rearing, substrate utilization, and nutritional value of housefly larvae. Our results indicated that nutrient loss in manure as a result of larvae growth was significant. Although the larva meal nutritional values we present may be specific for larvae raised in the particular source of cattle manure used in this study, they are still a good estimate for this species. Based on overall composition, larva meal is comparable to and possibly better than fishmeal in most respects. We particularly highlight the benefits of balanced amino acid composition, high monounsaturated fatty acids, and mineral content observed in housefly larva meal. These qualities affirm that larva meal could be a high-quality feed ingredient in livestock and aquaculture rations. However, aquaculture requires n-3 PUFAs in their diet ranging from 1–4% of dry diet depending on the species [67]. The finding that larva meal is not rich in n-3 PUFAs does not diminish its potential for use in this industry. Omega-3 fatty acids can be supplemented post-production from sustainable sources such as algae oil [68]. Based on the above results, future studies necessary to test the biological value of larva meal as a feed ingredient for specific species are well justified.

Larva meal can also meet certain novelty needs. Use of manure from organic farms can also allow for the production of an organic larva meal feed ingredient, something that has not been possible with the current ingredients in the aquaculture industry.

Overall, our results support that farming insect larvae has significant potential to develop as a novel mainstream agriculture operation and address several critical needs, but there is also urgent need for recognition of this approach. Federal research funding, private enterprise investments, and establishment of policy and regulations surrounding production and use of larva meal are necessary to compose a framework for commercial production and make this industry successful.
